# Fate of Antibiotic Resistant *Pseudomonas putida* and Broad Host Range Plasmid in Natural Soil Microcosms

**DOI:** 10.3389/fmicb.2019.00194

**Published:** 2019-03-01

**Authors:** Xiao-Ting Fan, Hu Li, Qing-Lin Chen, Yu-Sen Zhang, Jun Ye, Yong-Guan Zhu, Jian-Qiang Su

**Affiliations:** ^1^Key Laboratory of Urban Environment and Health, Institute of Urban Environment, Chinese Academy of Sciences, Xiamen, China; ^2^University of Chinese Academy of Sciences, Beijing, China; ^3^State Key Laboratory of Urban and Regional Ecology, Research Center for Eco-Environmental Sciences, Chinese Academy of Sciences, Beijing, China

**Keywords:** antibiotic resistance, horizontal gene transfer, conjugation, quantitative PCR, spread potential, flow cytometric sorting, phylogenetic analysis

## Abstract

Plasmid conjugation is one of the dominant mechanisms of horizontal gene transfer, playing a noticeable role in the rapid spread of antibiotic resistance genes (ARGs). Broad host range plasmids are known to transfer to diverse bacteria in extracted soil bacterial communities when evaluated by filter mating incubation. However, the persistence and dissemination of broad range plasmid in natural soil has not been well studied. In this study, *Pseudomonas putida* with a conjugative antibiotic resistance plasmid RP4 was inoculated into a soil microcosm, the fate and persistence of *P. putida* and RP4 were monitored by quantitative PCR. The concentrations of *P. putida* and RP4 both rapidly decreased within 15-day incubation. *P. putida* then decayed at a significantly lower rate during subsequent incubation, however, no further decay of RP4 was observed, resulting in an elevated RP4/*P. putida* ratio (up to 10) after 75-day incubation, which implied potential transfer of RP4 to soil microbiota. We further sorted RP4 recipient bacteria from the soil microcosms by fluorescence-activated cell sorting. Spread of RP4 increased during 75-day microcosm operation and was estimated at around 10^-4^ transconjugants per recipient at the end of incubation. Analysis of 16S rRNA gene sequences of transconjugants showed that host bacteria of RP4 were affiliated to more than 15 phyla, with increased diversity and shift in the composition of host bacteria. *Proteobacteria* was the most dominant phylum in the transconjugant pools. Transient transfer of RP4 to some host bacteria was observed. These results emphasize the prolonged persistence of *P. putida* and RP4 in natural soil microcosms, and highlight the potential risks of increased spread potential of plasmid and broader range of host bacteria in disseminating ARGs in soil.

## Introduction

The global increased antimicrobial resistance level in human pathogens has posed significant threat to human health (UNEP, 2017). Other than clinical settings, antibiotic resistance genes (ARGs) have been frequently detected with elevated diversity and abundance in soil (Zhu et al., 2013). Bacteria could become antibiotic resistant via gene mutation, or horizontal gene transfer (HGT) that would facilitate exchange of genetic information and contribute significantly to the evolution of bacteria (Boto, 2010; Soucy et al., 2015). HGT-mediated evolution would confer selective advantages to the host when facing environmental stress and thus promote the adaptation of bacteria under selective pressures (Daubin and Szollosi, 2016).

Plasmid conjugation is one of the most important mechanisms of HGT (Sorensen et al., 2005; Thomas and Nielsen, 2005), contributing to the wide spread of ARGs among environmental bacteria and human pathogens. Conjugative plasmids are often implicated in the rapid propagation and proliferation of ARGs (Dodd, 2012). These plasmids contain genes for the formation of pilus to transfer copies of themselves to other bacteria, enabling horizontal transfer of complete set of genes on the plasmid, including ARGs (Willetts and Wilkins, 1984; Willey et al., 2008). Furthermore, the transfer of diverse non-conjugative resistance plasmids can be facilitated by origin-of-transfer sequences of mobilizable plasmid (O’Brien et al., 2015). The massive use of antibiotics has resulted in high concentration of antibiotic residues in the environments, which may serve as a selection pressure that promote the dissemination of ARGs among environmental bacteria, including pathogenic species (Pruden et al., 2012; Guo et al., 2018). In addition, conjugative plasmids selected with antibiotics have been found to persist among bacterial populations or invade new strains even without antibiotics pressure (Dionisio et al., 2005).

The dissemination of ARGs caused by conjugation highly depends on the conjugation frequency and host range of plasmids. Broad host range plasmids are of high interest due to their transfer potency between phylogenetically distant hosts. Plasmid conjugation has been extensively studied in mating systems using typical pure strains as donors and recipients. Transconjugants can be quantified by colony counting on selective plates and the estimated transfer frequencies differs due to plasmids type, donor and recipient species, density ratio, mating time, and medium (Henschke and Schmidt, 1990; Modrie et al., 2010). However, culture-based screening of transconjugants can only partially determine the transfer frequency and identities of transconjugants in complex bacterial communities due to the limitation in culturing environmental bacteria (Elsas et al., 1989, 1990). Using fluorescent reporter genes to track plasmids made it possible to investigate the spread of resistance plasmids among environmental bacteria. To study the transfer of RP4 in activated sludge, the plasmid was marked with reporter gene *gfp* and detected by fluorescence microscopy, transfer frequencies were estimated in the range of 4 × 10^-6^ to 10^-5^ per recipient over a range of donor to recipient ratios (Geisenberger et al., 1999). Transconjugants obtained plasmids that marked with fluorescent genes can be sorted by fluorescence activated cell sorting (FACS) and identified by 16S rRNA gene sequencing (Musovic et al., 2006; Klumper et al., 2015).

Although these studies have expanded our understanding on plasmid conjugation, these mating experiments were generally conducted in relatively shorter mating period (4–72 h) (Sørensen et al., 2003; Wang et al., 2015b; Lin et al., 2016; Li et al., 2018) and optimized lab conditions that enabling sufficient cell-to-cell contact between donors and recipients (Musovic et al., 2010; Klumper et al., 2015, 2016; Li et al., 2018). Plasmid conjugation in soil could be affected by many factors including soil bacterial community composition, nutrients competition, and selective forces such as antibiotic and metal (Trevors et al., 1989; Elsas et al., 1990; Fox et al., 2008; Musovic et al., 2014; Klumper et al., 2016). However, current knowledge about plasmid conjugation in soil with complex bacterial communities is limited. The concentrations of donor bacteria and plasmid may significantly change after inoculation into soil, and the cell-to-cell contact between donors and recipients in soil is expected to decrease compared with filter mating systems, which could then affect plasmid conjugation in soil. In addition, retransfer of plasmid from transconjugants to the other bacterial populations may occur under extended incubation time.

To address these questions, soil microcosms were set up to investigate plasmid conjugation in soil. The aim of the study is to estimate the spread potential (SP) of plasmid and identify the phylogenetic affiliation of transconjugants in close to natural condition. *Pseudomonas putida* containing a broad host range plasmid tagged with green fluorescent protein (*gfp*) gene was used as donors and was inoculated into the soil microcosms. We tracked the abundance of both donor strains and plasmids in soil at intervals by qPCR. Transconjugants were collected with FACS and the 16S rRNA gene of transconjugants were amplified and sequenced.

## Materials and Methods

### Donor Strain and Plasmid

*Pseudomonas putida* KT2442 was used as donor, which was chromosomally tagged with *dsRed* and *lacI^q^* and harbored a broad host range IncP conjugative plasmid RP4 (Musovic et al., 2010). Plasmid RP4, containing resistance genes against ampicillin (Amp), kanamycin (Km), and tetracycline (Tc), was marked with *gfp* and a *lacI^q^* repressible promoter upstream of *gfp* gene, *gfp* expression was inhibited in donor strains, but when the plasmid transfer to a recipient, *gfp* expression is possible (both the donor strain and plasmid were kindly provided by Professor Barth F. Smets, Technical University of Denmark). The donor strains were grown aerobically under 180 rpm at 37°C overnight in LB medium supplemented with 100 μg ml^-1^ Amp, 50 μg ml^-1^ Km and 20 μg ml^-1^ Tc. The bacterial cells were harvested by centrifugation at 8500 *g* for 15 min and the cell pellets were washed twice and re-suspended in 0.9% sterile saline solution, the concentration of resulting donor inoculants was measured by colony-forming unit (CFU) counting.

### Soil Microcosms Set Up

Surface soil (sandy loam) was collected from a vegetable field in Xiamen, China, and was passed through a 2 mm sieve. Before inoculation, the original soil microbiota was extracted by Nycodenz density gradient separation (Burmolle et al., 2003; Eichorst et al., 2015) and the concentration of cells was 2.08 × 10^8^ cells g^-1^ dry soil which was determined by flow cytometry (FlowSight Imaging Flow Cytometer, Amnis Millipore, United States) after SYTO 9 staining. Soil microcosms were prepared in four replicate plastic pots by thoroughly mixing donor bacteria suspension into 500 g soil for each pot, providing a final donor strain density of 1.57 × 10^8^ CFU g^-1^ dry soil. The microcosms were incubated for 75 days in a greenhouse at room temperature (28°C in the night and 35°C in the daytime on average) and were regularly irrigated twice daily to keep the water content at 60% field water capacity. Control soil without donor inoculation were prepared and incubated at the same condition. Soil samples were collected from each pot on day 0, 1, 2, 3, 4, 5, 10, 15, 20, 30, 60, and 75 and were partitioned into two sub-samples: one stored in 4°C for Nycodenz extraction of bacterial cells and one stored in -20°C for total DNA extraction.

### DNA Extraction and qPCR

Soil DNA was extracted from 0.5 g of soil using FastDNA Spin kit for soil (MP Biomedical, Santa Ana, CA, United States). The concentrations of donor strain and plasmid were measured by quantification of *dsRed* and *gfp* genes, respectively. All quantifications were performed in triplicate using Roche 480 (Roche Molecular Systems Inc., Branchburg, NJ, United States). Real-Time PCR assays were performed in a total volume of 20 μl with 10 μl 2 × SYBR Premix ExTaq II (TaKaRa, Japan), 10 ng bovine serum albumin, 0.8 μl each primer (10 μM), 1 μl template DNA and 6.4 μl of nuclease-free PCR-grade water. The concentration of donor *P. putida* KT2442 in each sample was determined by quantifying a 702-bp region of *dsRed* gene using the primers dsRed-F (5^′^-ATATAGCATGCGGTCTTCCAAGAATGTTATCAA-3^′^) and dsRed-R (5^′^-CTCTCAAGCTTCCCGGGTTAAAGGAACAGATGGTGGCG-3^′^) (Tolker-Nielsen et al., 2000). Quantitative amplifications were performed with the following thermal cycles: 95°C for 5 min, followed by 40 cycles of 95°C for 50s, 60°C for 50 s, 72°C for 50 s, and a final extension at 72°C for 10 min. The concentration of plasmid RP4 was measured by quantifying a 89-bp region of *gfp* gene using the primers gfp-F (5^′^-GAAGATGGAAGCGTTCAA-3^′^) and gfp-R (5^′^-AGGTAATGGTTGTCTGGTA-3^′^) (Hale et al., 2015) with a thermal cycles as follows: 95°C for 5 min, followed by 40 cycles of 95°C for 40 s, 58°C for 30 s, 72°C for 30 s, and a final extension at 72°C for 10 min. Copy numbers of 16S rRNA gene were quantified by qPCR as described previously (Zhu et al., 2017). Details for preparation of standard curves of these genes were provided in Supplemental Method (Supplementary Figure S1). Control soil without inoculation was systematically incubated, sampled and was subjected to DNA extraction and gene quantification in parallel.

Means and standard deviations of qPCR results were calculated with Microsoft Office Excel 2016. Gene copy numbers were normalized by the weight of the dry soil. Concentrations of donor KT2442 and plasmid RP4 in soil microcosms were converted to lg(*C_t_/C_0_*) (Tolker-Nielsen and Molin, 2000), where *Ct* corresponds the concentration at time *t*, *C_0_* corresponds the concentration at time 0 (Al-Jassim et al., 2017). The phrase “spread potential” was selected to indicate the diffusion and proliferation of plasmid RP4 in the soil microbiota during incubation period. SP is considered as the ratio of transconjugants to recipients and was roughly estimated using the formula:

SPqPCR=(Cgfp/x−CdsRed)/(C16SrRNA/4−CdsRed)

SP_qPCR_ refers to the SP of RP4 estimated by qPCR, C is the concentration of gene determined by qPCR, presented as copies per gram dry soil. C*_gfp_*/x-C*_dsRed_* presents the number of transconjugants per gram of dry soil with assumption that every donor strain maintains plasmid. IncP plasmids are typically 1–3 copies per cell, here x refers to the copies of RP4 per cell and 1 or 3 was used for calculation, thus the range of SP_qPCR_ could be estimated. The average number of 16S rRNA-encoding genes per bacterial cell is currently estimated at 4.1 on the basis of the Ribosomal RNA Database (Klappenbach et al., 2001; Chen et al., 2017). C_16SrRNA_/4 presents the number of total bacteria per gram of dry soil.

### Flow Cytometry Sorting of Transconjugants

Bacteria from collected soil samples were extracted by Nycodenz density gradient separation and were re-suspended in PBS. Soil (1.5 g) was homogenized in 5 ml 1× phosphate buffer saline (PBS) supplemented with 0.5% (v/v) Tween-20 in triplicates. The mixtures were shaken for 30 min by a vortex in room temperature then added to the top of Nycodenz (HistoDenz, Sigma, United States) solution at 1.31 g ml^-1^ (6 g HistoDenz was dissolved in 10 ml sterile ultra-pure water) carefully, followed by centrifugation at 8500 *g* for 15 min. The upper PBS and bacteria layers were carefully collected, fivefold diluted (v/v) in PBS and centrifuged at 8500 *g* for 20 min (Musovic et al., 2010). The upper solution was discarded, the resulting pellet was re-suspended and centrifuged again until the bacteria were concentrated to 1 ml suspension.

Bacterial cells including indigenous soil bacteria (no fluorescence), transconjugants (green fluorescence) and donor strains (red fluorescence) were scaled by FlowSight Imaging Flow Cytometer (Amnis Millipore, United States). Transconjugants were sorted using S3e Cell Sorter (Bio-Rad, United States). The flow cytometer was equipped by 488 and 561 nm two-laser systems. GFP fluorescent signal was excited by 488 nm laser and collected in bandpass Filter1 (525/30 nm). DsRed fluorescent was excited by 561 nm and checked in Filter2 (586/25 nm). *Escherichia coli* K12 was prepared as a fluorescence negative control. *E. coli* K12 harbored RP4*::gfp* was used as *gfp*-positive control. Donor strain *P. putida* KT2442 (RP4*::gfp*) was prepared as *dsRed*-positive and *gfp*-negative control. According to the fluorescent intensity of control bacteria, the events with high green fluorescence intensity and no red fluorescence intensity were gated, sorted and collected to a 5 ml BD Falcon collection tubes based on the signal of FSC, SSC, Filter1, and Filter 2. Then the enriched *gfp*-positive events were subjected to an additional sorting under the purity mode to ensure that all collected particles were *gfp* positive.

To test the accuracy of FACS, green fluorescence of sorted transconjugants were confirmed by confocal laser scanning microscopy immediately. We also conducted a filter mating experiment using several pure bacterial strains as recipients. Mixtures of *Mangrovibacter yixingensis*, *Enterobacter tabaci*, *Enterobacillus tribolii*, *Pseudomonas aeruginosa* PAO1, *Burkholderia* sp., *Rhodococcus* sp., *Bacillus* sp., and *Staphylococcus* sp. with equal density were added to KT242/RP4 suspension at 1:1 cell ratio and filtered on 0.22 μm membrane filters (Millipore, United States). The filters with mating mixtures were placed on R2A agar and incubated at 37°C for 10 h. The mating cells were suspended in PBS for further flow cytometric sorting. Each green fluorescence-positive cell was sorted with single mode and collected in 100 μl PCR tubes. The plasmid marker gene *gfp* was amplified after whole genome amplification from 16 single transconjugants. GFP genes were 100% detected in each single cell and no donor strain or non-mating bacteria were sorted when verified by 16S rRNA gene amplification and sequencing.

The SP of RP4 estimated by flow cytometric sorting (SP_FCM_) was roughly calculated using the formula:

$$SP_{FCM}=gfp\;positive\;cells/(total\;cells\;-\;ds\;Red\;positive\;cells)$$

The plasmid-carrying donor strain was inoculated in the soil microcosms, which were incubated for 75 days. Plasmids transfer to recipients at different time and green fluorescent proteins in those transconjugant cells express with various levels. We only sorted out the events with higher fluorescent intensity to avoid the negative cells without RP4 being sorted.

### Amplification and Analysis of 16S rRNA Gene of Transconjugants

Sorted cells of each sample were concentrated in 100 μl PCR tubes and were subjected to three thermal cycles including 15 s in 100°C water and 45 s on ice. The V4–V5 region of 16S rRNA gene was amplified using primer 515F (5^′^-GTGCCAGCMGCCGCGG-3^′^) and 907R (5^′^-CCGTCAATCMTTTRAGTTT-3^′^) (Zhou et al., 2011). Each 2 × 50 μl PCR reactions contained 25 μl ExTaq (TaKaRa, Japan), 1 μl BSA of 20 μg ml^-1^, 1 μl each primer of 10 μM, 5 μl cell lysate as template and 17.5 μl nuclease-free PCR-grade water. The thermal program consisted of 95°C for 5 min, followed by 25 cycles of 95°C for 30 s, 58°C for 30 s, 72°C for 30 s, and a final extension at 72°C for 10 min. Non-template controls were included for detecting any contamination during PCR preparation (Chen et al., 2018). The amplicons were purified, quantified and sequenced on an Illumina Hiseq 2500 platform (Novogene, China) (Zhu et al., 2017). Raw pair-end reads were filtered to remove reads with low average quality scores and short reads (<300 nt). Sequences were processed with the Quantitative Insights Into Microbial Ecology (QIIME 1.9.1) pipeline (Caporaso et al., 2010; Edgar, 2010) as described previously (Zhu et al., 2017) for Operational Taxonomic Units (OTUs) clustering and taxonomic affiliation. Shannon index was generated to compare the level of bacterial diversity. Phylogenetic trees were constructed using MEGA 7 and iTOL^1^ (Letunic and Bork, 2016).

To determine the potential pathogen species in the transconjugant pools, all the high-quality 16S rRNA gene sequences from each sample were blasted against a bacterial pathogen database with an *E*-value < 1 × 10^-10^ and a sequence identity >99%. The bacterial pathogen database including 557 pathogenic species was constructed in our previous study (Chen et al., 2016).

## Results

### Fate of Donor Strain and Plasmid RP4

We quantified the marker genes *gfp* and *dsRed* to determine the concentrations of the plasmid RP4 and the donor strain KT2442, respectively. The fate of RP4 and KT2442 showed a similar decreased trend at the early stage of the microcosm operation, but differed at the late phase, resulting in a significantly increased RP4/KT2442 ratio during the incubation. The copy numbers of 16S rRNA gene in soil with KT2442/RP4 inoculation and control soil were at similar levels (3.3–4.9 × 10^9^ copies g^-1^ dry soil), and they both remained relatively stable during the incubation.

**FIGURE 1:**
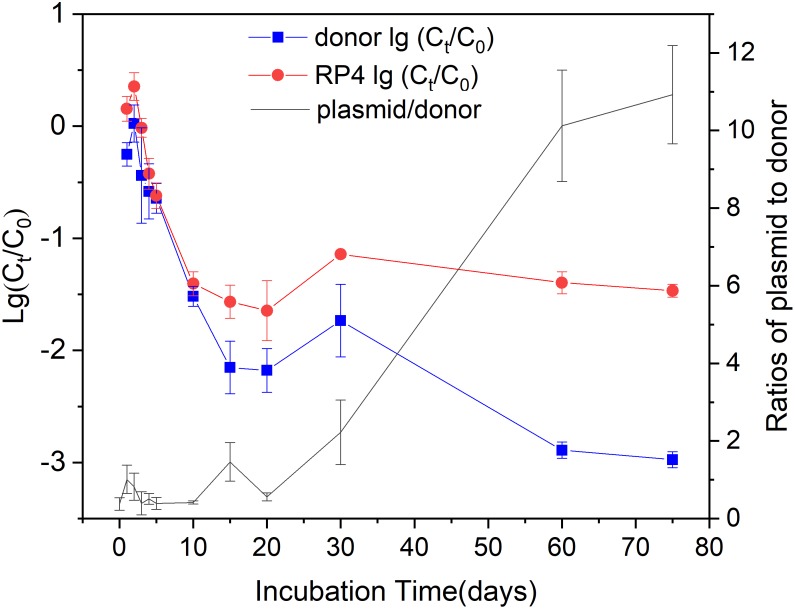
The decay of the donor and plasmid during incubation. Log values of the ratios of donor (plasmid) concentration at time *t* to the initial concentration were shown on the left *Y*-axis. The donor and plasmid concentrations were represented by the copy numbers of *dsRed* and *gfp* gene, respectively. The right *Y*-axis showed the ratios of plasmid RP4 to donor. Error bars represent standard error (SE) of sampling replicates on each time point (*n* = 3 or 4).

Both the fate of KT2442 and RP4 showed biphasic behaviors during the 75-days incubation. KT2442 was inoculated into the soil at an initial concentration of about 10^8^ CFU g^-1^ dry soil. They rapidly decreased 2.1 and 1.6 log to 10^6^ copies g^-1^ dry soil within 15 days incubation, respectively (Figure 1). After 15-days of incubation, the fate of KT2442 and RP4 followed different trends. KT2442 decayed at a significantly lower rate during subsequent incubation and persisted at a concentration around 10^5^ copies g^-1^ dry soil on the 60th and 75th day. However, no further decay of RP4 was observed, the concentration of RP4 persisted at a level of 10^6^ copies g^-1^ dry soil since the 10th day. Consequently, RP4/ KT2442 ratio significantly increased to around 10, indicating the occurrence of conjugation of RP4 to soil microbiota.

### Spread Potential of Plasmid RP4

We estimated the SP based on qPCR and flow cytometric sorting data, respectively. Copy numbers of *gfp* were consistently lower than that of *dsRed* until the 60th day, so it was not possible to estimate the SP in early soil microcosm samples using data of qPCR. SP_qPCR_ in samples of 60th day and 75th day was calculated using the formula described before, the results were 4.65 × 10^-4^–1.79 × 10^-3^ transconjugants per recipient (TC/R) and 3.63 × 10^-4^–1.42 × 10^-3^ TC/R, respectively.

Soil microbiota extracted from collected samples during incubation was analyzed and sorted by fluorescence activated cell sorter (FACS), including the early samples. The sorting results showed that RP4 had transferred to soil microbiota in the early stage of incubation. SP_FCM_ of samples on the 5th day was 7.5 × 10^-5^ TC/R, while it increased to 2.5 × 10^-4^ TC/R on the 75th day. Only cells with strong green fluorescence signal were collected by flow cytometry, so the transconjugant numbers could be underestimated.

### Phylogenetic Analysis of Transconjugant Pools

**FIGURE 2:**
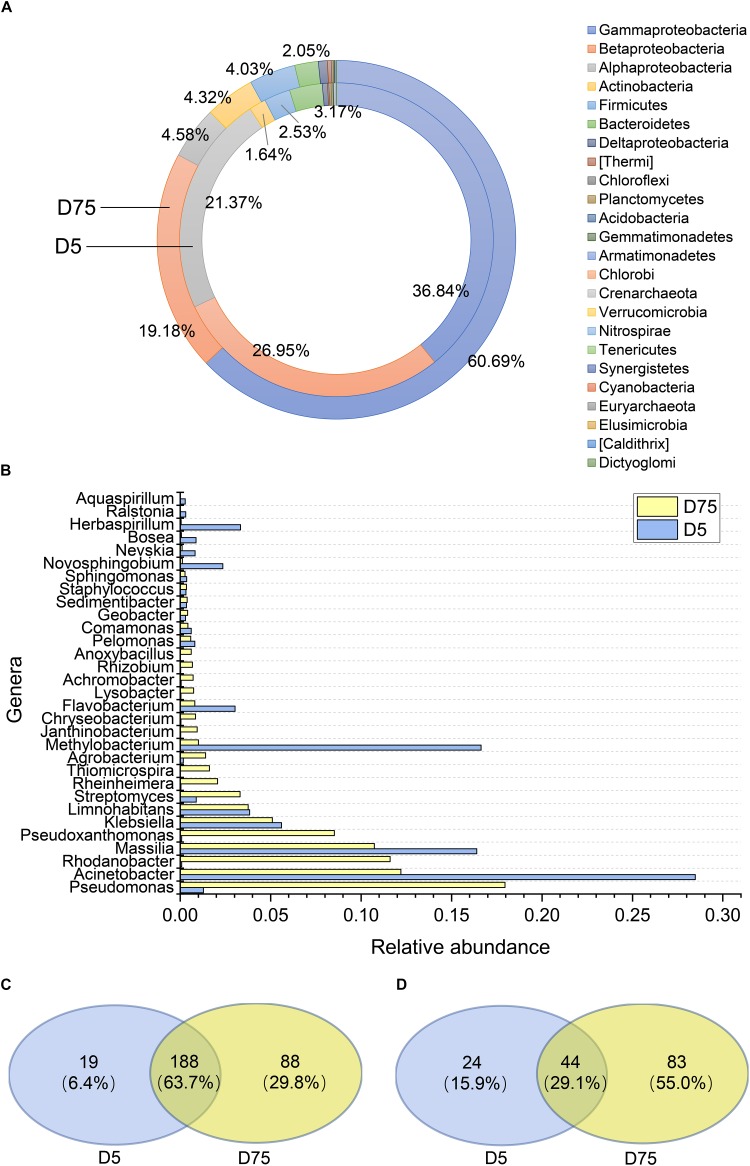
Composition of sorted transconjugant pools on day 5 and day 75. **(A)** Phylum distribution. **(B)** The relative abundance of the top 20 genera on day 5 or day 75. **(C)** Venn diagram showing the number of shared and unique genera between D5 and D75. **(D)** Venn diagram showing the number of shared and unique core OTUs that contributed 95% sequences among D5 and D75 samples.

Transconjugants were sorted and subjected to taxonomic identification based on 16S rRNA gene sequences. The results indicated that transconjugants covered most of the major bacterial phyla, and the diversity of transconjugants differed at different time points. More than 300 transconjugant OTUs over 15 phyla were acquired from D5 samples. *Proteobacteria* was the most dominant phylum, contributing 85.7% of the transconjugant pools, followed by *Bacteroidetes* (3.17%), gram-positive *Firmicutes* (2.53%), and *Actinobacteria* (1.64%) (Figure 2A). The diversity of transconjugants significantly increased in D75 samples, in which three times more transconjugant OTUs were identified and affiliated to 20 phyla. Shannon index of D5 transconjugants was 2.38, while it was 4.41 of D75 transconjugant pools. *Proteobacteria*, *Bacteroidetes*, *Firmicutes*, and *Actinobacteria* were still the dominant phyla, contributing 95.6% of total transconjugants (Figure 2A). The relative abundance of *Actinobacteria* increased to 4.32% including 30 genera, while there were only 21 genera with lower abundance in D5 samples. The proportion of Gammaproteobacteria increased from 36.8 to 60.7% and Alphaproteobacteria decreased from 21.4 to 4.58%.

Nine of the top 20 genera of D75 were shared with D5 (Figure 2B), they were *Acinetobacter*, *Methylobacterium*, *Massilia*, *Klebsiella*, *Limnohabitans*, *Flavobacterium*, *Pseudomonas*, *Streptomyces*, and *Pelomonas*, among which 7 genera were affiliated to *Proteobacteria*. The relative abundance of shared top genera changed over time. *Acinetobacter* and *Massilia* occupied high proportions in both transconjugant pools. *Methylobacterium* was the second dominant genus of D5 transconjugants, with a relative proportion of 16.6%, while it decreased to 1.01% in D75. On the contrary, *Pseudomonas* was the most dominant genus of D75, contributed to 18.0% of transconjugant pool when excluding out the donor strain species *P. putida*. While it was 1.28% in D5. *Pseudomonas*, *Acinetobacter*, and *Staphylococcus* were detected with relative high frequency. Opportunistic pathogenic species such as *Acinetobacter baumannii*, *Aeromonas veronii*, *Enterobacter cloacae*, *Pseudomonas mendocina*, and *Staphylococcus aureus* were identified in the further sequence analysis (Supplementary Table S1). Transconjugant pools between D5 and D75 shared 63.7% genera (Figure 2C), indicating that plasmid transferred into most genera of the recipients at the early invasion stage. However, a total of 88 unique genera were detected in D75 samples, indicating significantly increased diversity of transconjugants. In addition, only 19 unique genera were detected in D5 samples, implying that these recipients may lose RP4 in subsequent incubation. Analysis of core OTUs of transconjugants accounting for 95% of the sequences indicated 127 OTUs in D75 and 66 OTUs of D5 (Figure 2D), among which, only 44 OTUs were shared between D5 and D75 samples.

**FIGURE 3:**
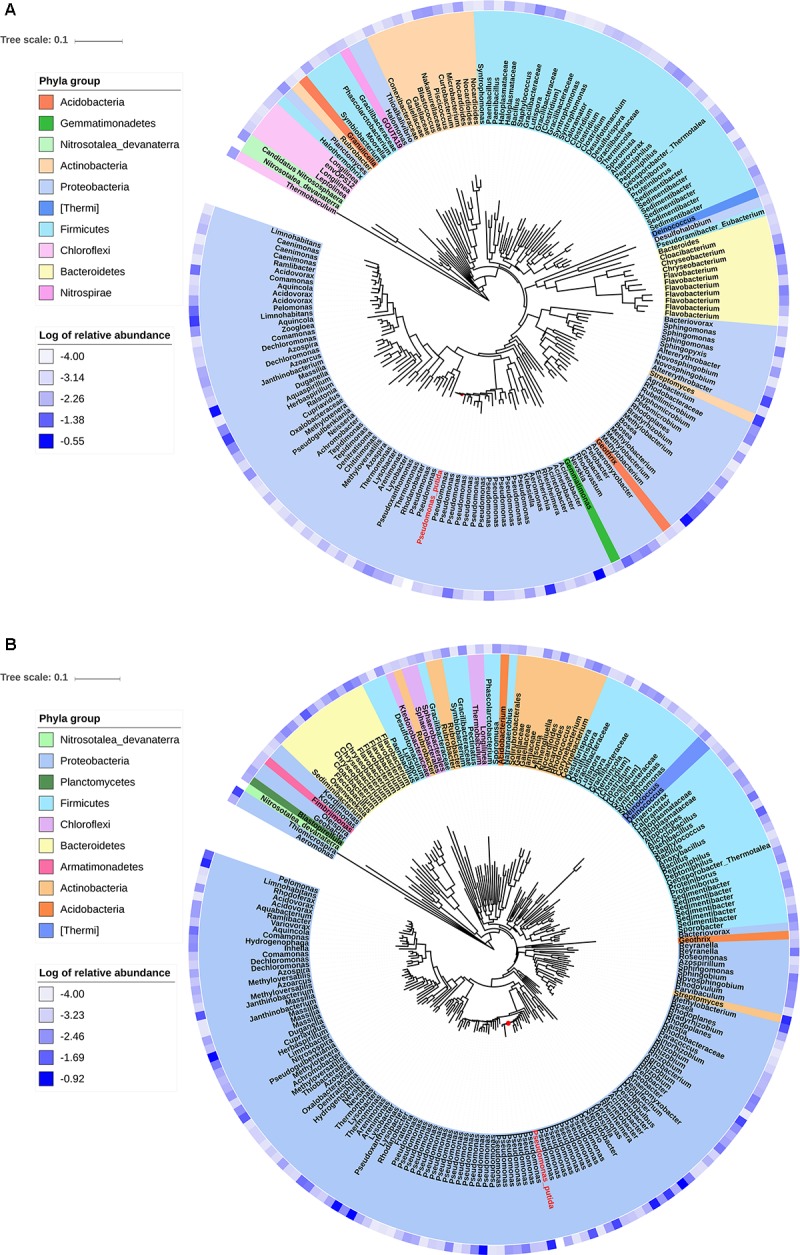
Phylogenetic tree showing the identified OTUs that contributed more than 0.01% in the transconjugant pools. **(A)** Showing transconjugants on D5. **(B)** Showing transconjugants on D75. The 16S rRNA gene sequence of *Pseudomonas putida* (donor strain) was shown in red letters and *Nitrosotalea devanaterra* (distant relative species to most of the transconjugants) was imported as the reference. The blue gradient circle at the periphery of the tree represents log of relative abundance of the OTU in the transconjugant pools.

Phylogenetic trees were constructed using the sequences of OTUs with relative abundance more than 0.01% in the transconjugant pools. The phylogenetic distance between donor strain *P. putida* and the transconjugants was shown, as well as the relative abundance of OTUs (Figure 3). RP4 transferred from *P. putida* to a broad range of recipients including phylogenetic distant group. However, the advantage of *Proteobacteria* was outstanding, with more than half of branches were identified to be *Proteobacteria*. Remarkably, much more branches identified to be *Pseudomonas* appeared in tree of D75, indicating the advantage of intra-genus transfer.

## Discussion

Human activities, including land application of manure, sewage sludge and wastewater, are providing direct routes for disseminating antibiotic resistant bacteria and genes to soil (Zhu et al., 2018). The spread of ARGs in soil is closely associated with HGT, however, studies on the HGT in soil are challenging due to the complexity of HGT process in soil (Williams-Nguyen et al., 2016). In this study, we use quantitative PCR and an established fluorescent bioreporter system to estimate the SP and delineate the taxonomic affiliation of recipient populations of a plasmid carrying multiple ARGs in soil. Occurrence of HGT need physical contact between donor and recipient populations, thus the population density of donor strain in soil is an important parameter for HGT (Martinez et al., 2015).

Using *P. putida* KT2442 harboring RP4 as a model strain, we thus investigated the dynamics of donor in a vegetable soil microcosm. We observed a biphasic decay behavior of both *P. putida* KT2442 and RP4. This biphasic decay had been well documented in *P. putida*, *E. coli* and pathogens (Brouwer et al., 2017; Mantilla-Calderon and Hong, 2017). The concentrations of *dsRed* and *gfp* were average 1.3 × 10^8^ and 4.3 × 10^7^ copies g^-1^ dry soil after initial spiking event, and they slightly increased at first 2 days after inoculation, which might be due to the division of donor strains and was in accordance with previous studies (Elsas et al., 1989; Henschke and Schmidt, 1990). Concentration of *P. putida* KT2442 rapidly decreased but a significantly reduced decay rate was observed after 15 days, and the copy number of *dsRed* maintained at 10^5^ copies g^-1^ dry soil at the late stage of incubation. *P. putida* is frequently detected in natural soil and this result suggested that *P. putida* KT2442 survived and established in soil for a relatively longer period, which may highly affect the persistence and proliferation of plasmid, as well as the transfer of plasmid (Turner et al., 1998).

Plasmid RP4 showed a similar decay at the early stage of incubation, however, no further decay was observed after 15 day. The copy numbers of *gfp* plateaued at average 10^6^ copies g^-1^ dry soil during the following incubation period, resulting an escalated plasmid/donor ratio that peaked at 10 on day 60. The plasmid decay kinetics may mainly be affected by three factors, the lysis of host bacteria and subsequent release of plasmid, the stability of plasmid in host bacteria, and plasmid transfer between host and recipient populations. Previous reports have shown that IncP plasmid is stable in *Pseudomonas* (Shintani et al., 2006; Yano et al., 2007), thus cellular decay of *P. putida* KT2442 may drive the kinetic of RP4 at the early stage of incubation. Though the copies of RP4 decreased rapidly and were lower to the concentrations of KT2442 at the early stage, plasmid transfer potentially occurred during the period, which was confirmed by flow cytometric sorting and analysis of transconjugants. It was reported that transconjugants appeared immediately after introduction of *E. coli* C600(RP4) or *P. putida* BH(RP4) into soil microcosms (Inoue et al., 2009). The donor bacteria were added with a cell ratio at 1:1 to indigenous soil bacteria, which was a relative high level although this ratio had been reported in previous studies (Elsas et al., 1990; Kozdrój, 2008; Lin et al., 2016; Stalder and Top, 2016). We conducted the experiment with a cell ratio at 1:1 for two reasons. Firstly, a higher conjugation frequency was observed when the mating densities of donor and recipients were equal according to a early study (Qiu et al., 2012). Secondly, added bacteria would decay rapidly when inoculated into microcosms (Elsas et al., 1989; Inoue et al., 2009; Bonot and Merlin, 2010), a high initial concentration of donors may be conducive to maintaining the donor cells and the subsequent analysis of plasmid transfer in this long-term experiment. Further experiments should be conducted with a gradient of donor cells for better understanding the transfer of plasmid in soil.

An established fluorescent bioreporter approach was adopted to estimate the SP of RP4 in soil, which was 7.5 × 10^-5^ TC/R on day 5 and 2.5 × 10^-4^ TC/R on day 75. The SP significantly increased during incubation, the longer incubation period with donor in soil microcosms may partially contribute to the spread of plasmid in this study, which provide sufficient time for plasmid transfer between donors and recipients, or even between transconjugants and recipients. The composition of recipient bacterial community is another factor that could significantly affect the SP of plasmid (Lopatkin et al., 2016). For example, the transfer frequency that estimated via a 48-h filter mating experiment using the same donor strain and plasmid with sewage sludge microbiota ranged from 3 to 50 conjugation events per 100000 cells (Li et al., 2018). Other factors, including antibiotics (Subbiah et al., 2011), nanomaterials (Qiu et al., 2012), metals (Klumper et al., 2016), UV irradiation (Lin et al., 2016), or ionic liquid (Wang et al., 2015a) may also affect the plasmid transfer in soil. Further studies should be conducted to investigate the effect of multiple factors on plasmid transfer and persistence in soil. The overall conjugation dynamics were determined by conjugation efficiency and propagation of transconjugants (Lopatkin et al., 2016). Discriminating the contribution of these two processes is challenging in soil. Nevertheless, our results provided an overall estimation of the proportion of host bacteria of plasmid in soil microbiota, which is essential for evaluating the potential role of plasmid transfer in mediating the dissemination of ARGs in soil after long-term exposure to antibiotic resistant bacteria.

Phylogenetic analysis of sorted transconjugants indicated that RP4 disseminated to a broad range of hosts, which had been documented in previous reports, with *Proteobacteria* being the predominant phylum (Klumper et al., 2015, 2016; Li et al., 2018), followed by *Bacteroidetes*, *Firmicutes*, and *Actinobacteria*. Gammaproteobacteria, known as the dominant hosts for the broad host range plasmid (Suzuki et al., 2010; Norberg et al., 2011), occupied 41.75% of total transconjugants in day 75. The most dominant genera in transconjugant pool of D75 including *Pseudomonas*, *Acinetobacter*, and *Rhodanobacter* were all from this class. However, it was observed that the phylogenetic diversity increased during the incubation with 88 unique genera were detected in day 75, implying that the plasmid may transfer to a broader range of taxa upon extended exposure time in soil than we expected. As the concentrations of *P. putida* KT2442 and RP4 were at roughly the same level from day 15 to 30, the increased diversity of transconjugant pools could be attributed, at least in part, to the plasmid transfer between transconjugants and recipient populations. A significant shift of transconjugant pool composition was also observed, for examples, 11 of the top 20 genera in day 75 were different from those in day 5 samples (Figure 3B). *Rhodovastum* (0.15%)/*Sphingopyxis* (0.13%) (Alphaproteobacteria), and *Planctomyces* (0.17%, *Planctomycetes*) were detected on day 5, but were not recovered on day 75. These data indicated that RP4 transiently transferred to members of these genera but they lost the plasmid during the following incubation, probably due to the fitness cost of the plasmid or the plasmid cannot replicate in these recipients. Nevertheless, the existing of transient host bacteria could aid in the survival and persistence of plasmid (Yano et al., 2013) in soil, and consequently contribute to the dissemination of ARGs in soil.

## Author Contributions

J-QS and Y-GZ designed the experiments. X-TF and HL performed the microcosm experiments and qPCR. X-TF performed the flow cytometric sorting. Q-LC, Y-SZ, and JY conducted the phylogenetic analysis. All authors contributed to data analysis. X-TF and J-QS wrote the manuscript with comments from all authors.

## Conflict of Interest Statement

The authors declare that the research was conducted in the absence of any commercial or financial relationships that could be construed as a potential conflict of interest.

## Footnotes

1 http://itol.embl.de/
