# Supplementary material for: Fate of Antibiotic Resistant Pseudomonas putida and Broad Host Range Plasmid in Natural Soil Microcosms
Source: Front Microbiol. 2019 Mar 1;10:194. doi: 10.3389/fmicb.2019.00194 (PMC6407330; doi:10.3389/fmicb.2019.00194)
Supplement: Supplementary file 1 [file Data_Sheet_1.docx]

**Supplementary information** for

**Fate of antibiotic resistant *Pseudomonas putida* and broad host range plasmid in natural soil microcosms**

*Xiao-Ting Fan^1,2^, Hu Li ^1^, Qing-Lin Chen**^1^, Yu-Sen Zhang^1,2^, Jun Ye^1^, Yong-Guan Zhu^1,2,3^, Jian-Qiang Su^1,*^*

*^1^Key Laboratory of Urban Environment and Health, Institute of Urban Environment, Chinese Academy of Sciences, Xiamen, China*

*^2^University of Chinese Academy of Sciences, Beijing, China*

*^3^State Key Laboratory of Urban and Regional Ecology, Research Center for Eco-Environmental Sciences, Chinese Academy of Sciences, Beijing, China*

* *Correspondence: Jian-Qiang Su, Key Laboratory of Urban Environment and Health, Institute of Urban Environment, Chinese Academy of Sciences, Xiamen, China. E-mail:* [*jqsu@iue.ac.cn*](mailto:jqsu@iue.ac.cn)

1. Standard curves

Standard curves were established before qPCR analyses on a Roche LightCycler480 instrument (Roche, USA) to quantify *gfp*, *dsRed* and 16S rRNA genes. After normal PCR amplification, *amplicons of gfp*, *dsRed* and 16S rRNA genes were recovered, purified and ligated onto a pMD^TM^19-T vector (TaKaRa, Japan) and transformed to *E.coli* DH5α according to manufacturer’s instructions (TaKaRa, Japan). Positive clones were screened by plating selection with ampicillin, followed by PCR and sequencing to verify cloning of the target genes. Plasmids carrying target genes were extracted using a plasmid extraction kit (Tiangen, China) and were used as the standards for qPCR. The concentration and quality of the plasmid were determined by spectrophotometric analysis and agarose gel electrophoresis. Eight-point standard curves for qPCR were generated using 10-fold serial dilutions of the plasmid carrying target genes. Efficiency values were close to 2 and the error values were lower to 0.2 for all standard curves. According to the standard curves, the Ct values of samples were used to calculate the gene copies of *gfp*, *dsRed* and 16S rRNA genes. Standard curves of quantitative PCR for *dsRed,* *gfp* and 16S rRNA genes (Six-point curve) are shown below.


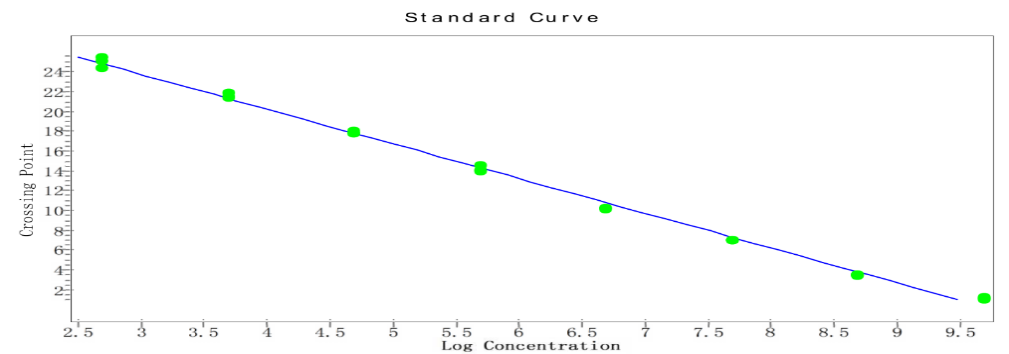


Target gene: *dsRed*

Error: 0.148

Efficiency: 1.928

Slope: -3.509


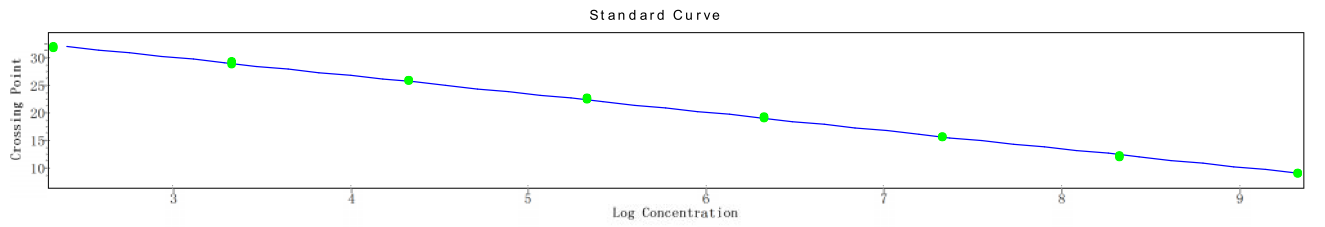


Target gene: *gfp*

Error: 0.0688

Efficiency: 2.003

Slope: -3.315


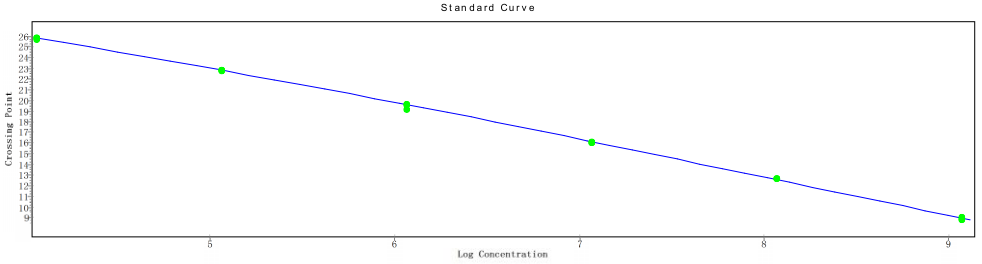


Target gene: 16S rRNA

Error: 0.00918

Efficiency: 1.969

Slope: -3.399

Figure S1 Standard curves of quantitative PCR for *dsRed,* *gfp* and 16S rRNA genes.

Table S1 Blast results of representative sequences that were annotated to be opportunistic pathogens

| No. | Sequence | reference pathogenic species | Score | Identities |
| --- | --- | --- | --- | --- |
| 1 | TAATACAGAGGGTGCGAGCGTTAATCGGATTTACTGGGCGTAAAGCGTACGTAGGCGGCTTATTAAGTCGGATGTGAAATCCCTGAGCTTAACTTAGGAATTGCATTCGATACTGGTGAGCTAGAGTATGGGAGAGGATGGTAGAATTCCAGGTGTAGCGGTGAAATGCGTAGAGATCTGGAGGAATACCGATGGCGAAGGCAGCCATCTGGCCTAATACTGACGCTGAGGTACGAAAGCATGGGGAGCAAACAGGATTAGATACCCTGGTAGTCCATGCCGTAAACGATGTCTACTAGCCGTTGGGGCCTTTGAGGCTTTAGTGGCGCAGCTAACGCGATAAGTAGACCGCCTGGGGAGTACGGTCGCAAGACTA | *Acinetobacter baumanii ZW85-1_NC023028* | 684 | 99% |
| 2 | TAATACGGAGGGTGCAAGCGTTAATCGGAATTACTGGGCGTAAAGCGCACGCAGGCGGTTGGATAAGTTAGATGTGAAAGCCCCGGGCTCAACCTGGGAATTGCATTTAAAACTGTCCAGCTAGAGTCTTGTAGAGGGGGGTAGAATTCCAGGTGTAGCGGTGAAATGCGTAGAGATCTGGAGGAATACCGGTGGCGAAGGCGGCCCCCTGGACAAAGACTGACGCTCAGGTGCGAAAGCGTGGGGAGCAAACAGGATTAGATACCCTGGTAGTCCACGCCGTAAACGATGTCGATTTGGAGGCTGTGTCCTTGAGACGTGGCTTCCGGAGCTAACGCGTTAAATCGACCGCCTGGGGAGTACGGCTGCAAGGTTA | *Aeromonas veronii B565_NC015424.1* | 689 | 99% |
|  |  |  |  |  |
| 3 | TAATACGGAGGGTGCAAGCGTTAATCGGAATTACTGGGCGTAAAGCGCACGCAGGCGGTCTGTCAAGTCGGATGTGAAATCCCCGGCCTCAACCTGGGAACTGCATTCGAAACTGGCAGGCTAGAGTCTTGTAGAGGGGGGTAGAATTCCAGGTGTAGCGGTGAAATGCGTAGAGATCTGGAGGAATACCGGTGGCGAAGGCGGCCCCCTGGACAAAGACTGACGCTCAGGTGCGAAAGCGTGGGGAGCAAACAGGATTAGATACCCTGGTAGTCCACGCCGTAAACGATGTCGACTTGGAGGTTGTGCCCTTGAGGCGTGGCTTCCGGAGCTAACGCGTTAAGTCGACCGCCTGGGGAGTACGGCCGCAAGGTTA | *Enterobacter cloacae Nr.3_Y17665.1* | 689 | 99% |
| 4 | TAATACGAAGGGTGCAAGCGTTAATCGAAATTACTGGGCGTAAAGCGCGCGTAGGTGGTTCGTTAAGTTGGATGTGAAAGCCCCGGGCTCAACCTGGGAACTGCATCCAAAACTGGCGAGCTAGAGTACGGTAGAGGGTGGTGGAATTTCCTGTGTAGCGGTGAAATGCGTAGATATAGGAAGGAACACCAGTGGCGAAGGCGACCACCTGGACTGATACTGACACTGAGGTGCGAAAGCGTGGGGAGCACACAGGATTAGATACCCTGGTAGTCCACGCCGTAAACGATGTCAACTAGCCGTTGGAATCCTTGAGATTTTAGTGGCGCAGCTAACGCATTAAGTTGACCGCCTGGGGAGTACGGCCGCAAGGTTA | *Pseudomonas mendocina ymp_NC009439.1* | 684 | 99% |
| 5 | TAATACGTAGGTGGCAAGCGTTATCCGGAATTATTGGGCGTAAAGCGCGCGTAGGCGGTTTTTTAAGTCTGATGTGAAAGCCCACGGCTCAACCGTGGAGGGTCATTGGAAACTGGAAAACTTGAGTGCAGAAGAGGAGAGTGGAATTCCATGTGTAGCGGTGAAATGCGCAGAGATATGGAGGAACACCAGTGGCGAAGGCGACTTTCTGGTCTGTAACTGACGCTGATGTGCGAAAGCGTGGGGATCAAACAGGATTAGATACCCTGGTAGTCCACGCCGTAAACGATGAGTGCTAAGTGTTAGGGGGTTTCCGCCCCTTAGTGCTGCAGCTAACGCATTAAGCACTCCGCCTGGGGAGTACGACCGCAAGGTTG | *Staphylococcus aureus GSA-A16_ JN315155.1* | 697 | 100% |
|  |  |  |  |  |
